# Supplementary material for: Multi-parameter photon-by-photon hidden Markov modeling
Source: Nat Commun. 2022 Feb 22;13:1000. doi: 10.1038/s41467-022-28632-x (PMC8863987; doi:10.1038/s41467-022-28632-x)
Supplement: Supplementary file 3 — Reporting Summary [file 41467_2022_28632_MOESM3_ESM.pdf]

nature research

Corresponding author(s): Paul David Harris, Eitan Lerner

Last updated by author(s): Feb 3, 2022

Reporting Summary

Nature Research wishes to improve the reproducibility of the work that we publish. This form provides structure for consistency and transparency in reporting. For further information on Nature Research policies, see our Editorial Policies and the Editorial Policy Checklist.

Statistics

For all statistical analyses, confirm that the following items are present in the figure legend, table legend, main text, or Methods section.

n/a	Confirmed	
☒	☐	The exact sample size (*n*) for each experimental group/condition, given as a discrete number and unit of measurement
☒	☐	A statement on whether measurements were taken from distinct samples or whether the same sample was measured repeatedly
☒	☐	The statistical test(s) used AND whether they are one- or two-sided<br>*Only common tests should be described solely by name; describe more complex techniques in the Methods section.*
☒	☐	A description of all covariates tested
☐	☒	A description of any assumptions or corrections, such as tests of normality and adjustment for multiple comparisons
☐	☒	A full description of the statistical parameters including central tendency (e.g. means) or other basic estimates (e.g. regression coefficient) AND variation (e.g. standard deviation) or associated estimates of uncertainty (e.g. confidence intervals)
☒	☐	For null hypothesis testing, the test statistic (e.g. *F*, *t*, *r*) with confidence intervals, effect sizes, degrees of freedom and *P* value noted<br>*Give P values as exact values whenever suitable.*
☒	☐	For Bayesian analysis, information on the choice of priors and Markov chain Monte Carlo settings
☒	☐	For hierarchical and complex designs, identification of the appropriate level for tests and full reporting of outcomes
☒	☐	Estimates of effect sizes (e.g. Cohen's *d*, Pearson's *r*), indicating how they were calculated

Our web collection on statistics for biologists contains articles on many of the points above.

Software and code

Policy information about availability of computer code

Data collection	Single molecule data was collected with common acquisition software (labView v7.1, Symphotime 2.4.4874 or ISS VistaVision version 4.2.095)
Data analysis	Raw data was converted into photon-HDF5 format using the python package phconvert v0.8.2. Data analysis was conducted with FRETBursts, using the version available at https://github.com/harripd/FRETBursts/tree/315c60d3791aa93cf2ec6e880003174c8192fc88 .<br>mpH2MM analysis was conducted with the python package H2MM_C v0.8.2 https://github.com/harripd/H2MMpythonlib/tree/7386da8576de820643a146225f5bb63cf4ecbcc4 .<br>Simulations of diffusion were conducted with PyBroMo v0.8.1 (https://github.com/tritemio/PyBroMo/tree/075dec0eb5747a9edf9a28eb2ec487319e440ade).<br>FRETBursts code is available at https://github.com/harripd/FRETBursts.

For manuscripts utilizing custom algorithms or software that are central to the research but not yet described in published literature, software must be made available to editors and reviewers. We strongly encourage code deposition in a community repository (e.g. GitHub). See the Nature Research guidelines for submitting code & software for further information.

Data

Policy information about availability of data

All manuscripts must include a data availability statement. This statement should provide the following information, where applicable:

- Accession codes, unique identifiers, or web links for publicly available datasets
- A list of figures that have associated raw data
- A description of any restrictions on data availability

All data used in this study, including all photon-HDF5 file containing smFRET data, and files generated by PyBroMo simulations are available on zenodo:

Field-specific reporting

Please select the one below that is the best fit for your research. If you are not sure, read the appropriate sections before making your selection.

- [x] Life sciences
- [ ] Behavioural & social sciences
- [ ] Ecological, evolutionary & environmental sciences

For a reference copy of the document with all sections, see nature.com/documents/nr-reporting-summary-flat.pdf

Life sciences study design

All studies must disclose on these points even when the disclosure is negative.

Sample size	Sample size (bursts) is determined by the duration of acquisition. Durations are chosen so that thousands of bursts are acquired.
Data exclusions	No data was excluded from analysis.
Replication	All attempts at replication were successful. Technical repeats produced consistent data. We describe our setup and software in detail to allow recreation of our results.
Randomization	Data does not need to be randomized as observations rely on single molecules diffusing through the confocal spot. This provides sufficient randomization during acquisition.
Blinding	No blinding is necessary as the subjects of this study are single molecules.

Reporting for specific materials, systems and methods

We require information from authors about some types of materials, experimental systems and methods used in many studies. Here, indicate whether each material, system or method listed is relevant to your study. If you are not sure if a list item applies to your research, read the appropriate section before selecting a response.

Materials & experimental systems

n/a	Involved in the study
x	Antibodies
x	Eukaryotic cell lines
x	Palaeontology and archaeology
x	Animals and other organisms
x	Human research participants
x	Clinical data
x	Dual use research of concern

Methods

n/a	Involved in the study
x	ChIP-seq
x	Flow cytometry
x	MRI-based neuroimaging
